# A two-year randomized clinical trial of bulk-fill and ion-releasing composites with universal adhesives in class V carious lesions

**DOI:** 10.1007/s00784-026-06852-5

**Published:** 2026-04-11

**Authors:** Hoda Saleh Ismail, Hanan Ahmed Nabil Soliman, Ashraf Ibrahim Ali, Ahmed Gamal Raghip, Eman H. Albelasy

**Affiliations:** 1 Conservative Dentistry Department, Faculty of Dentistry, Mansoura University, Algomhoria Street, PO Box 35516, Mansoura, Egypt; 2 Conservative Department, Faculty of Dentistry, Kafr Al Sheikh University, Kafr Al Sheikh, Egypt; 3 Faculty of Oral and Dental Medicine, Restorative Dentistry Department, Alsalam University, Tanta, Egypt

**Keywords:** Class V restorations, Clinical performance, Fluoride-releasing universal adhesive, Marginal adaptation

## Abstract

**Objectives:**

To evaluate the two-year clinical performance of Class V restorations in carious cervical lesions placed with bulk-fill resin composite or ion-releasing composite, combined with either fluoride-free or fluoride-releasing universal adhesives.

**Materials and methods:**

In this double-blind, randomized clinical trial, 140 Class V restorations were placed using four restorative systems: bulk-fill composite with fluoride-free adhesive, bulk-fill composite with fluoride-releasing adhesive, ion-releasing composite with fluoride-free adhesive, and ion-releasing composite with fluoride-releasing adhesive. Restorations were placed under rubber-dam isolation using selective enamel etching. Clinical evaluations were performed at baseline, 6 months, one year, and two years using periodontal indices and FDI criteria. Data were analyzed using Kruskal–Wallis, Friedman, Mann–Whitney U, and Wilcoxon signed-rank tests (α = 0.05).

**Results:**

Recall rates were 96.4% at 6 months, 89.3% at 1 year, and 83.5% at 2 years. Periodontal parameters showed no significant differences among groups. No significant differences were observed between restorative systems for any FDI functional, biological, or esthetic criteria at any evaluation point. All restorations remained clinically acceptable, with no loss of retention, fractures, or secondary caries throughout the two-year follow-up. Marginal adaptation scores remained stable and comparable among all groups.

**Conclusions:**

All four restorative systems demonstrated comparable and favorable clinical performance over two years. The use of fluoride-releasing adhesives or ion-releasing composites did not confer additional clinical benefits under the conditions of this trial.

**Clinical relevance:**

Both the tested bulk-fill resin composite and ion-releasing composite deliver predictable short-term performance in Class V restorations, without additional benefit from fluoride-releasing components.

**Supplementary Information:**

The online version contains supplementary material available at 10.1007/s00784-026-06852-5.

## Introduction

In the past ten years, significant progress has been achieved in the development of resin composites to tackle various clinical difficulties. Enhanced techniques for bulk placement, innovative filler formulations, and streamlined adhesion protocols have made the application process more convenient [1]. Despite these improvements, certain challenges such as technique sensitivity, polymerization shrinkage, and the absence of antibacterial characteristics have persisted [2]. Furthermore, secondary caries and bulk fractures continue to be the primary causes of failures associated with resin composites [1].

Restorations in cervical lesions frequently fail for reasons that differ from those seen in other cavity classes. Studies on clinical performance indicate that loss of retention, deterioration of marginal adaptation, and marginal staining are among the most frequently reported issues in cervical restorations rather than catastrophic bulk fractures, which are more typical in posterior load-bearing restorations [3]. Meta-analyses of long-term clinical trials on cervical restorations have shown a wide range of outcomes, with average retention losses around 10–12%, marginal discoloration reported in approximately 24–28% of restorations, and deterioration of marginal integrity in a substantial proportion of cases, while secondary caries is detected much less frequently [4]. These outcomes underscore the importance of adhesive performance and marginal sealing for cervical restorations, given their limited macro-mechanical retention and proximity to dentinal substrates [4].

Studies have revealed that thicker biofilms tend to gather around resin composite restorations as compared to glass ionomer restorations [5]. Further in vivo research on the plaque has demonstrated that the presence of lactic acid-producing bacteria is significantly greater around resin composite restorations compared to both amalgam and glass ionomer restorations [6]. As a measure to prevent the development of secondary caries, fluoride-releasing materials that exhibit remineralization and/or antibacterial properties have gained traction in recent times [7].

There are now new ion-releasing composites that are commercially available and claim to have bioactive properties. These composites are a recent addition to the existing range of ion-releasing composites and are said by manufacturers to release a significant amount of ions, apart from fluoride, to help with remineralization around restorations [8]. While laboratory studies have demonstrated that ion-releasing restorations have a reduced risk of secondary caries, it is unclear how restorative materials relate to the development of secondary caries in clinical settings [9].

Fluoride-containing restorative materials have been developed to provide an antibacterial effect, thereby contributing to the prevention of secondary caries at cavity margins [10, 11]. However, the ability of these materials to release and recharge fluoride is a crucial factor that can affect their cariostatic effect [11]. While fluoride can be added to the material’s formulation, the anti-cariogenic potential generated by fluoride integration may not be adequate to completely prevent secondary caries [10, 11]. The concentration of fluoride released and the duration for which it is released varies depending on the materials, with a greater amount typically being released initially followed by decline [12]. Recent in vitro evidence suggests that fluoride‑releasing restorative materials exhibit fluoride release and associated inhibition of lesion progression adjacent to restorations [13], but systematic review data indicate that the overall clinical effectiveness of fluoride release alone in preventing secondary caries remains limited or inconsistent [14].

Clinical evidence regarding the performance of cervical restorations remains limited, particularly when compared with the extensive body of laboratory data available. Most clinical information on Class V restorations originates from studies conducted on non-carious cervical lesions, while comparatively few investigations have focused on restorations placed in carious cervical lesions [3, 4]. Meta-analyses and long-term clinical trials evaluating cervical restorations in non-carious cervical lesions have consistently reported loss of retention, marginal discoloration, and deterioration of marginal integrity as the most frequent clinical outcomes, whereas the occurrence of secondary caries has generally been low [3, 4]. In contrast, clinical studies specifically addressing restorative performance in carious cervical lesions remain scarce, despite the biological and clinical differences between carious cervical lesions and non-carious cervical lesions, particularly the presence of caries-affected dentin and their association with patient-related caries susceptibility [15]. Caries activity has been identified as a relevant factor influencing the development and progression of carious cervical lesions and may affect the long-term clinical behavior of restorative materials, especially with respect to marginal integrity and secondary caries development [16]. Although ion-releasing and fluoride-containing restorative materials have been proposed to provide additional protection against caries, recent systematic reviews and network meta-analyses have reported inconsistent clinical benefits regarding secondary caries prevention, particularly in restorations with adequate marginal sealing placed under controlled clinical conditions [16, 17]. Consequently, there is a clear need for well-designed randomized clinical trials that specifically evaluate contemporary restorative systems in carious cervical lesions while using standardized clinical evaluation criteria [15].

Given the available data and the recognized role of fluoride in preventing secondary caries, it is worth asking whether fluoride-releasing adhesives and ion-releasing restorative materials can effectively preserve marginal adaptation and thereby reduce marginal discoloration and secondary caries. Furthermore, it is essential to examine whether the combination of a fluoride-releasing adhesive with an ion-releasing restorative material could enhance their anti-cariogenic action, considering the limitations of the ion-releasing properties of these restorative materials when used with adhesives, as reported in the literature. Based on the abovementioned data, the current study aimed to evaluate and compare the 2-year clinical performance of Class V restorations in carious cervical lesions restored using various restorative systems with resin composite and ion-releasing composites, utilizing fluoride-free or fluoride-releasing universal adhesives. The null hypotheses tested were: (a) There is no difference in the clinical performance (periodontal parameters and functional, biological, and esthetic outcomes according to FDI criteria) among the different restorative systems at the same evaluation period. (b) There is no difference in the clinical performance of each restorative system among the different evaluation periods over the two-year follow-up.

## Materials and methods

### Restorative materials utilized

This clinical trial assessed two universal adhesives: a non-fluoride-releasing universal adhesive (Tetric N‑Bond Universal, Ivoclar Vivadent, Amherst, NY, USA) and a fluoride-releasing universal adhesive (CLEARFIL TRI-S BOND Universal Quick, Kuraray Noritake Dental Inc, Okayama, Japan). Additionally, two composites were evaluated: a nanohybrid bulk-fill resin composite (Tetric PowerFill, Ivoclar Vivadent, Amherst, NY, USA) and an ion-releasing composite (Activa BioActive-Restorative, Pulpdent Corp., Watertown, MA, USA). Refer to Table 1 for a detailed description of these materials.

**Table 1:** Materials used in the study

Material	pH	Type	Manufacturer	Composition	Application technique	Lot number
Tetric *N*‑Bond Universal	2.5	Non-fluoride releasing universal adhesive	Ivoclar Vivadent, Amherst, NY, USA	HEMA, Bis-GMA, D3MA, MDP, ethanol, water, methacrylate-modified polyacrylic acid, silicon dioxide, camphorquinone, ethyl *p*-dimethyl aminobenzoate, 2-dimethyl aminoethyl methacrylate.	1. Apply bond with rubbing action for 20 s. 2. Disperse with oil- and moisture-free compressed air until a glossy, immobile film layer results 3. Light cure for 10 s.	Z04LY6
CLEARFIL TRI-S BOND Universal Quick	2.3	Fluoride-releasing universal adhesive	Kuraray Noritake Dental Inc, Okayma, Japan	10-MDP, Bis-GMA, 2-HEMA, hydrophilic amide monomers, colloidal silica, silane coupling agent, sodium fluoride, dl camphorquinone, ethanol, water	1. Apply with a rubbing motion 2. Blowing mild air for more than 5 s 3. Light cure for 10 s	B30324
Tetric PowerFill (^IV^A)	Nanohybrid bulk fill resin composite		Ivoclar Vivadent	Bis-GMA, Bis-EMA, UDMA, Bis-PMA, DCP. Fillers; Barium glass, Ytterbium, Trifluoride, Copolymer, Mixed Oxide (SiO_2_/ZrO_2_) (79 wt%, 53–54 vol%)		Z02P3N
Activa BioActive-Restorative (A2)	Ion-releasing composite		Pulpdent Corp., Watertown, MA, USA	Diurethane dimethacrylate, bis (2-(methacryloyloxy) ethyl) phosphate, barium glass, ionomer glass, sodium fluoride, colorants, polyacrylic acid/maleic acid copolymer Filler loading: 56 wt%		220916

### Study design and blinding

This study adhered to the guidelines outlined in the Consolidated Standards of Reporting Trials Statement (CONSORT) [18]. A parallel, double-blind experimental design was employed, ensuring blinding for both patients and examiners. Based on the materials described in Sect. 2.1, the study included four restorative systems according to the combination of composite type and adhesive fluoride content: (1) bulk-fill composite with non-fluoride-releasing adhesive (negative control), (2) bulk-fill composite with fluoride-releasing adhesive, (3) ion-releasing composite with non-fluoride-releasing adhesive, and (4) ion-releasing composite with fluoride-releasing adhesive (positive control).

### Sample size calculation

For this study, the sample size was calculated based on the primary clinical outcome of restoration retention, which is considered a key indicator of clinical performance in Class V restorations. The calculation was based on data from a previous clinical study that compared the two-year performance of resin composite and resin-modified glass ionomer restorations in cervical lesions [19]. The formula used to calculate the number of subjects (n) in each group was [20]: n = [(Z α/2 + Z β)^2^ x {(p1 (1-p1) + (p2 (1-p2))}]/(p1 – p2)^2^.

An appropriate sample size of 28 restorations was estimated, with a significance level of 5% (*p* < 0.05) and a power of 80%. Considering a potential dropout rate of 20%, the final sample size was set at 35 restorations per group.

### Patient selection

In this specific research, adult individuals seeking dental treatment at the Operative Department Clinic of the Faculty of Dentistry were included. The main goal was to evaluate 140 Class V restorations. A single operator assessed the volunteers to determine if they met the inclusion criteria. This assessment involved gathering the patient’s medical and dental history, conducting a clinical examination, and performing a radiographic examination (periapical) when needed. All included cavities represented primary carious cavitated cervical lesions, and teeth with existing restorations, secondary caries, or replacement restorations were excluded from the study. Before participating, patients were provided with comprehensive details about the study procedures and gave their written consent [21]. Ethical approval was obtained beforehand for the study, which was also registered on ClinicalTrials.gov.

### Eligibility criteria

The study included volunteers of both genders aged between 25 and 45 years. Participants were required to present with a minimum of one and a maximum of two primary cavitated cervical carious lesions located exclusively on the buccal surface of upper or lower posterior teeth.

Eligible lesions had not extended beyond half of the buccal surface in the occlusogingival direction, did not cross the two proximal line angles, and did not extend more than 1 mm apical to the gingival margin. The final cavity depth was required to extend axially into dentin but not exceed 1.5 mm. All included lesions were active cavitated cervical carious lesions requiring operative intervention, while inactive or arrested lesions were excluded. Clinically, the lesions corresponded to International Caries Detection and Assessment System (ICDAS II) code 5 (distinct cavitation with visible dentin). Lesions with extensive structural breakdown (ICDAS II code 6) were excluded.

The selected teeth were required to be vital, confirmed using an electric pulp tester (DY310, Denjoy, Hunan, China), insensitive to percussion, free of spontaneous pain, and without radiographic evidence of periapical or furcation radiolucency on preoperative radiographs. The cervical carious lesions were required to present with an enamel margin occlusally and either an enamel or dentin margin cervically. In addition, the affected teeth had to have adjacent teeth with normal alignment, and the opposing teeth had to be sound and free of restorations.

To ensure clinical standardization, only patients presenting with one or two localized active cavitated cervical carious lesions requiring operative treatment were included. Eligible participants were required to be in good general health, with no history of allergies to dental materials or medications, and willing to attend the scheduled recall appointments.

Patients were excluded if they had active periodontal disease, xerostomia, or extremely poor oral hygiene with generalized untreated caries activity. Pregnant or nursing individuals were also excluded. In addition, individuals using fluoride-containing products (including fluoride toothpastes, mouthrinses, gels, or professional fluoride applications) were excluded to minimize the confounding effects of external fluoride sources and to allow a more accurate evaluation of the clinical performance of the tested fluoride-releasing and non–fluoride-releasing restorative systems. Patients using desensitizing agents, undergoing orthodontic treatment, or exhibiting severe bruxism resulting in more than 50% tooth wear were also excluded.

Following enrollment, all participants were instructed to use a fluoride-free toothpaste and to maintain regular tooth-brushing throughout the study period. One week prior to the restorative procedure, all patients received professional prophylaxis performed by a periodontist to ensure optimal plaque control and gingival health.

The baseline characteristics of the participants and the outcomes of the treated cavities are presented in Supplementary Tables S1 and S2.

### Random sequence generation and allocation concealment

During the study, each patient underwent the placement of either one or two Class V restorations in carious cervical lesions using one of the four specified restorative systems. The choice of restorative material for each patient was randomly assigned using a computer-generated list of random numbers created with the Microsoft^®^ Excel program. A team member not participating in the clinical trial compiled this list. The outcomes of the random sequence were sealed in envelopes. Upon a patient meeting the inclusion criteria and being enrolled, an envelope was opened, assigning the patient to one of the four groups. No blinding procedures were implemented beyond this stage.

### Clinical procedures

#### Cavity preparation and isolation procedures

Before commencing the restorative procedures, the patients received local anesthesia with 1.8 mL of a solution containing 2% lidocaine and 1:25,000 phenylephrine (Novocol 100, SS White Dental Manufacturing, Brazil) to minimize discomfort. A braided epinephrine impregnated retraction cord (size 00, Gingi-Pak MAX Z-Twist, Gingi-Pak Pharmaceutical, CA, USA) was placed in the buccal gingival sulcus at the beginning of the procedure. The initial cavity preparation was carried out using a high-speed handpiece (W&H, RC-90RM, Austria) with a round medium grit diamond bur (801 − 010, Komet, Brasseler GmbH & Co. KG, Lemgo, Germany) and water coolant. The preparation design was determined by the decay’s extent. During this phase, all cavity walls were maintained with nearly butt joint margins featuring slightly rounded line angles. A new bur was used after five cavity preparation. The retraction cord was removed, and a sterilized Teflon piece was placed in the gingival sulcus before applying rubber dam isolation. The operative area was isolated with a rubber dam kit (KSK DENTECH, Tokyo, Japan) and high suction. Next, selective carious excavation was performed based on clinical (tactile) evaluations of any detected carious lesions on the axial wall [22]. This was carried out using a low-speed handpiece with a tungsten round bur (H1.204.014, Komet) under water cooling and a hand excavator (Maillefer, Dentsply DeTrey GmbH, Konstanz, Germany) until reaching firm dentin. A 1-mm-wide bevel was prepared on the occlusal enamel margins of all cavities at an inclination of 45^°^, using a long cylindrical diamond bur (837 L-014, Komet). An accurate assessment of the cavity was then conducted, excluding any cavity with an axial depth exceeding 1.5 mm. Additionally, any cavity dimension that was more than half the occlusal length or extended beyond the proximal line angles was also excluded. Finally, the type of the gingival margin was recorded to be either on enamel or dentin.

### Restorative procedures

The occlusal, proximal and gingival enamel margins (in case of cases with enamel gingival margins) of the cavities in all groups were treated with 37% phosphoric acid (N-Etch, Ivoclar Vivadent, NY, USA) for 15 s, followed by a 15-second water rinse and gentle drying with oil-free air, avoiding desiccation. Next, the universal adhesive specific to each group was applied to all cavity surfaces, air-thinned, and then light cured using a light emitting diode (LED) curing light (Elipar Deep Cure, 3 M ESPE, St. Paul, MN, USA) at a power of > 1000 mW/cm^2^ as per the manufacturer’s recommendation. The curing light operation was monitored every five cases with a radiometer (Demetron L.E.D. Radiometer, Kerr Corp., Orange, CA, USA) to ensure proper functioning.

The cavities were subsequently filled with the corresponding restorative material using a bulk technique. In the case of the nanohybrid bulk fill composite group, the material was applied to the cavity with a plastic instrument (Artman Instruments, GA, USA). For the ion-releasing composite group, the material was dispensed using long, thin-tipped applicators provided by the manufacturer, applying gentle and steady pressure until the cavity was slightly overfilled. All materials were cured with the same LED light curing device mentioned earlier according to the manufacturer’s instructions [10 s for the nanohybrid bulk fill composite, 20 s for the ion-releasing composite (after 25 s for self-cure)].

Once the rubber dam was removed, the restorations were finalized, ensuring any visible overhangs were eliminated using a tapered round long finishing diamond stone (8850-014, Komet). Special attention was given to ensuring no visible overhangs or rough restoration surfaces at the gingival margins. The surfaces were polished using a low-speed handpiece with silicon carbide-impregnated points and cups (KENDA AG, Vaduz, Liechtenstein) with water cooling, following the recommended sequence.

All preparation and restoration procedures were conducted by a single operator using magnification (4× loupes; Amtech, Wenzhou, China) and LED headlight illumination. Digital photography was employed to document preoperative conditions and all steps of the restoration process. Patients were scheduled for a follow-up appointment one week later for baseline measurements, and then at 6 months, and at one and two years’ post-restoration for evaluations and reinforcement of oral hygiene practices.

### Evaluation

The evaluation procedures were carried out in a randomized order by two trained examiners who were kept unaware of the allocation and restoration processes. Periodontal and clinical assessments of all restorations were conducted one week post the restorative procedures (baseline) and then at intervals of six months, one and two years. Intraoral clinical images were taken during each recall period, and all findings were documented utilizing a standardized paper case report form. Parameters necessitating visual inspection were examined using a magnifying dental loupe (2.5×) positioned at a working distance of 40 cm, with ample illumination from an attached light source. Following the assessment, the case reports were submitted to a member of the research team to maintain the blinding of the evaluators regarding group allocations during subsequent follow-up evaluations.

### Examiner training

Prior to commencing the evaluation for the study, the examiners underwent two training sessions separated by several hours, focusing on ten similar clinical cases. Intra- and inter-examiner agreements on the evaluation numbers and scores were determined using the intra-class correlation coefficient (ICC) and Cohen’s kappa coefficient (κ). A criterion of > 90% intra- and inter-examiner agreements was set as a requirement for the calibration of all evaluations before the assessment process commenced.

### Primary outcome measures

The primary outcome of this study was the clinical performance of Class V restorations over time. Throughout each assessment interval (one week, six months, one and two years), a set of parameters was evaluated and documented. The two examiners evaluated all periodontal parameters in a consistent sequence, with a 45-minute interval between each examiner’s assessment on the same day. A manual graduated periodontal Williams probe (Zoll-Dental, Niles, IL, USA) was utilized by both examiners to record readings to the nearest millimeter. The periodontal parameters included the plaque index (PI), gingival index (GI) following the Löe and Silness method [23, 24], bleeding on probing index (BOP) per Ainamo and Bay [25], and probing depth (PD).

Restoration evaluations for all teeth adhered to the updated FDI criteria [26] and were ranked correspondingly. The assessment categories encompassed functional properties (fracture and retention, marginal adaptation, form and contour), biological properties (caries at the restoration margin, tooth defects at the restoration margins, and postoperative hypersensitivity), esthetic properties (marginal staining). The study evaluated postoperative sensitivity by subjecting the restored tooth surface to a 2-second cold-air stimulus using a triple syringe positioned approximately 2.0 cm away. This stimulus was administered while neighboring teeth were isolated with cotton rolls, and the pain level was gauged utilizing the Visual Analogue Scale.

### Secondary outcome measures

The secondary outcomes included selected esthetic and patient-centered parameters, namely surface luster and texture and patient’s view, evaluated according to the updated FDI criteria.

### Statistical analysis

All data was meticulously organized into tables, coded, and then subjected to statistical analysis utilizing SPSS software (version 20, IBM, Chicago, IL, USA). The distribution of gingival margin location (enamel or dentin) among the four restorative material groups was assessed using a chi-square test of independence to confirm that the gingival margin substrate was comparable across groups.

For the PI, GI, and FDI scoring data, comparisons between different restorative systems within the same evaluation period were performed using the Kruskal–Wallis and Mann–Whitney *U* tests. To assess the baseline data of these parameters against other evaluation periods within the same restorative system group, the Friedman and Wilcoxon signed rank tests were employed.

In the case of the BOP index, the chi-square test was utilized to compare different systems within the same evaluation period. Additionally, the McNemar test was employed to compare data from different evaluation periods for the same restorative system.

Regarding PD, different restorative system groups within the same evaluation period were compared using the Kruskal–Wallis test. To compare baseline data with other evaluation periods for the same parameter within the restorative system group, repeated measures analysis of variance (ANOVA) along with post hoc tests were conducted.

All comparisons were conducted at a significance level of 5%. Intra- and inter-examiner agreement during examiner calibration and after each evaluation period were evaluated using the ICC and κ statistics.

## Results

All the planned restorative procedures were executed without any deviations.

### Recall rates

**Fig. 1:**
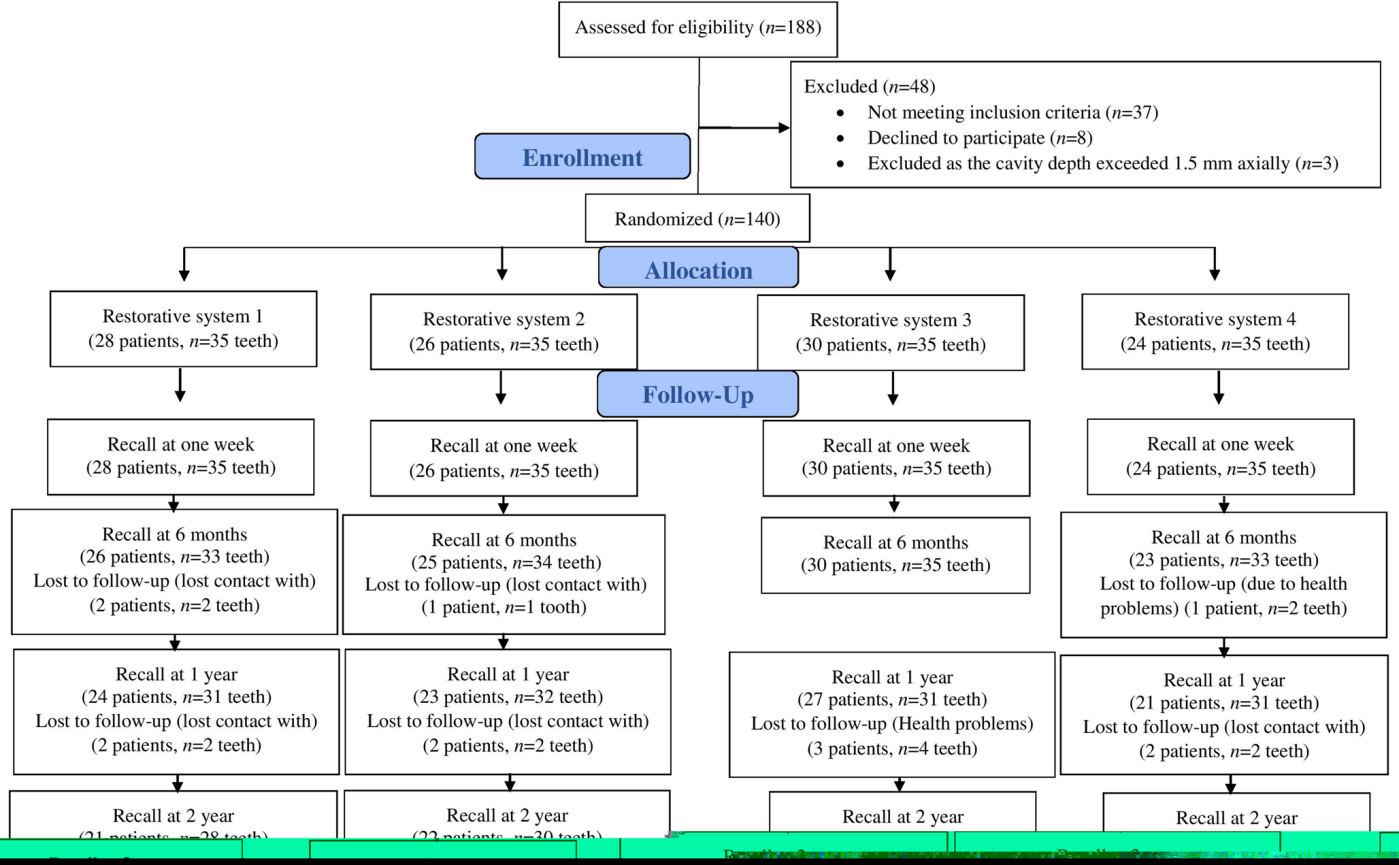
Shows the CONSORT flow diagram, which details the number of restorations allocated for intervention, the number lost, and the number of restorations available for analysis at each follow-up

The follow-up rates were perfect at 100% for the baseline (one week), 96.4% for the six-month, 89.3% for the one-year, and 83.5% for the two-year follow-ups (Fig. 1).

### Agreement among examiners and within examiner during follow-up visits

The levels of consistency between examiners ranged from ICC 0.90 to 0.94 and κ 0.89 to 0.92 across various follow-up sessions. Intra-examiner agreement levels varied from ICC 0.94 to 0.98 and κ 0.92 to 0.97.

### Results of the primary outcome

The distribution of the gingival margin location (enamel or dentin) was not significantly different among the four restorative material groups (*p* = 0.57), indicating that the type of marginal tissue was comparable across groups.

The results of all evaluated periodontal parameters for the various restorative systems across different evaluation periods are presented in Tables 2, 3, 4 and 5. When comparing different restorative systems within the same evaluation periods (using Kruskal-Wallis test results for PI, GI and PD data, as well as chi-square test for BOP data), no statistically significant differences were found between the systems.

**Table 2:** Results of gingival index [GI] (%) of different restorative systems’ groups over two years’ follow-up period

Restorative system	Follow-up period	GI score		
		0	1	2
BF/F−	Baseline	19 (67.9%)	8 (28.6%)	1 (3.6%)
	6-month P1 < 0.001	6 (21.4%)	18 (64.3%)	4 (14.3%)
	One year P1 = 0.022, P2 = 0.166	9 (32.1%)	17 (60.7%)	2 (7.1%)
	Two year P1 = 0.015, P2 = 0.439, P3 = 0.157	9 (32.1%)	15 (53.6%)	4 (14.3%)
BF/F+	Baseline	16 (53.3%)	12 (40%)	2 (6.7%)
	6-month P1 = 0.011	5 (16.7%)	22 (73.3%)	3 (10%)
	One year P1 = 0.593, P2 = 0.008	14 (46.7%)	14 (46.7%)	2 (6.7%)
	Two year P1 = 0.439, P2 = 0.013, P3 = 0.317	14 (46.7%)	15 (50%)	1 (3.3%)
IR/F−	Baseline	14 (46.7%)	15 (50%)	1 (3.3%)
	6-month	9 (30%)	18 (60%)	3 (10%)
	One year	13 (43.3%)	15 (50%)	2 (6.7%)
	Two year	14 (46.7%)	15 (50%)	1 (3.3%)
IR/F+	Baseline	16 (55.2%)	12 (41.4%)	1 (3.4%)
	6-month P1 = 0.029	9 (31%)	17 (58.6%)	3 (10.3%)
	One year P1 = 0.317, P2 = 0.109	14 (48.3%)	13 (44.8%)	2 (6.9%)
	Two year P1 = 0.132, P2 = 0.248, P3 = 0.157	12 (41.4%)	15 (51.7%)	2 (6.9%)

*BF* bulk-fill resin composite, *IR* ion-releasing composite, *F−* fluoride-free universal adhesive, *F+* fluoride-releasing universal adhesive

*P1* significance relative to Baseline, *P2* significance relative to 6-month, *P3* significance relative to one year between different follow-up periods of each base material separately. Significance at *p* < 0.05. For system 3, the Friedman test revealed no statistically significant difference between the evaluation periods (*p* = 0.237); thus, no multiple comparisons were performed.

**Table 3:** Results of bleeding on propping index [BOP] (%) of different restorative systems’ groups over two years’ follow-up period

Restorative system	Follow-up period	BOP score	
		0	1
**BF/F−**	Baseline	20 (71.4%)	8 (28.6%)
	6-month P1 = 0.289	16 (57.1%)	12 (42.9%)
	One year P1 = 0.125, P2 = 1	16 (57.1%)	12 (42.9%)
	Two year P1 = 0.700, P2 = 0.727, P3 = 0.625	14 (50%)	14 (50%)
**BF/F+**	Baseline	25 (83.3%)	5 (16.7%)
	6-month P1 = 0.031	19 (63.3%)	11 (36.7%)
	One year P1 = 0.727, P2 = 0.344	23 (76.7%)	7 (23.3%)
	Two year P1 = 0.344, P2 = 0.774, P3 = 0.625	21 (70%)	9 (30%)
**IR/F−**	Baseline	25 (83.3%)	5 (16.7%)
	6-month P1 = 0.125	21 (70%)	9 (30%)
	One year P1 = 0.016, P2 = 0.453	18 (60%)	12 (40%)
	Two year P1 = 0.031, P2 = 0.687, P3 = 1	19 (63.3%)	11 (36.7%)
**IR/F+**	Baseline	24 (82.8%)	5 (17.2%)
	6-month P1 = 0.125	20 (69%)	9 (31%)
	One year P1 = 0.031, P2 = 0.687	18 (62.1%)	11 (37.9%)
	Two year P1 = 0.016, P2 = 0.453, P3 = 1	17 (58.6%)	12 (41.4%)

*BF* bulk-fill resin composite, *IR* ion-releasing composite, *F−* fluoride-free universal adhesive, *F+* fluoride-releasing universal adhesive

*P1* significance relative to Baseline, *P2* significance relative to 6-month, *P3* significance relative to one year between different follow-up periods of each base material separately. Significance at *p* < 0.05

**Table 4:** Results of plaque index [PI] (%) of different restorative systems’ groups over two years’ follow-up period

Restorative system	Follow-up period	PI score		
		0	1	2
BF/F−	Baseline	23 (88.1%)	5 (17.9%)	0 (0%)
	6-month P1 < 0.001	10 (35.7%)	18 (64.3%)	0 (0%)
	One year P1 < 0.001, P2 = 0.011	3 (10.7%)	24 (85.7%)	1 (3.6%)
	Two years P1 < 0.001, P2 = 0.007, P3 = 0.317	3 (10.7%)	23 (85.7%)	2 (7.1%)
BF/F+	Baseline	23 (67.7%)	7 (23.3%)	0 (0%)
	6-month P1 < 0.001	7 (23.3%)	23 (67.7%)	0 (0%)
	One year P1 < 0.001, P2 = 0.414	5 (16.7%)	25 (83.3%)	0 (0%)
	Two years P1 < 0.001, P2 = 0.096, P3 = 0.083	2 (6.7%)	28 (93.3%)	0 (0%)
IR/F−	Baseline	22 (73.3%)	8 (26.7%)	0 (0%)
	6-month P1 = 0.004	13 (43.3%)	16 (53.3%)	1 (3.3%)
	One year P1 < 0.001, P2 < 0.001	2 (6.7%)	25 (83.3%)	3 (10%)
	Two years P1 < 0.001, P2 < 0.001, P3 = 0.180	1 (3.3%)	24 (80%)	5 (16.7%)
IR/F+	Baseline	21 (72.4%)	8 (27.6%)	0 (0%)
	6-month P1 < 0.001	11 (37.9%)	17 (58.6%)	1 (3.4%)
	One year P1 < 0.001, P2 = 0.003	3 (10.3%)	24 (82.8%)	2 (6.9%)
	Two years P1 < 0.001, P2 = 0.003, P3 = 0.180	2 (6.9%)	23 (79.3%)	4 (13.8%)

*BF* bulk-fill resin composite, *IR* ion-releasing composite, *F−* fluoride-free universal adhesive, *F+* fluoride-releasing universal adhesive

*P1* significance relative to Baseline, *P2* significance relative to 6-month, *P3* significance relative to one year between different follow-up periods of each base material separately. Significance at *p* < 0.05

**Table 5:** Results of probing depth (PD) of different restorative systems’ groups over two years’ follow-up period

Follow-up period Restorative system	Baseline	6-month	One year	Two years
BF/F−	2.32 ± 0.17	2.42 ± 0.18 P1 = 0.03	2.62 ± 0.31 P1 < 0.001, P2 = 0.01	2.67 ± 0.25 P1 < 0.001, P2 < 0.001, P3 = 1
BF/F+	2.36 ± 0.26	2.47 ± 0.26 P1 = 0.11	2.56 ± 0.33 P1 = 0.009, P2 = 0.39	2.61 ± 0.32 P1 = 0.001, P2 = 0.04, P3 = 1
IR/F−	2.21 ± 0.29	2.39 ± 0.27 P1 = 0.002	2.51 ± 0.32 P1 < 0.001, P2 = 0.07	2.56 ± 0.35 P1 < 0.001, P2 = 0.03, P3 = 1
IR/F+	2.30 ± 0.24	2.45 ± 0.23 P1 = 0.02	2.62 ± 0.38 P1 < 0.001, P2 = 0.04	2.71 ± 0.35 P1 < 0.001, P2 = 0.004, P3 = 0.19

*BF* bulk-fill resin composite, *IR* ion-releasing composite, *F−* fluoride-free universal adhesive, *F+* fluoride-releasing universal adhesive

*P1* significance relative to Baseline, *P2* significance relative to 6-month, *P3* significance relative to 12-month between different follow-up periods of each base material separately. Significance at *p* < 0.05

Regarding PI, the Kruskal-Wallis test data comparisons for baseline (*p* = 0.825), 6-month (*p* = 0.556), one-year (*p* = 0.282), and two-year data (*p* = 0.192) showed no significant differences. Similarly, for GI, the Kruskal-Wallis test data comparisons for baseline (*p* = 0.479), 6-month (*p* = 0.646), one-year (*p* = 0.68), and two-year data (*p* = 0.539) did not reveal any significant variances. In the case of PD, the Kruskal-Wallis data comparisons for baseline (*p* = 0.07), 6-month (*p* = 0.465), one-year (*p* = 0.44), and two-year data (*p* = 0.247) showed no statistically significant differences.

Across all evaluated periodontal parameters, the baseline data were lower than the data from subsequent evaluation periods for all tested restorative systems (Tables 2, 3, 4 and 5). Moreover, no statistically significant differences were observed between the data from one and two years for any of the evaluated parameters in the tested restorative systems.

**Fig. 2:**
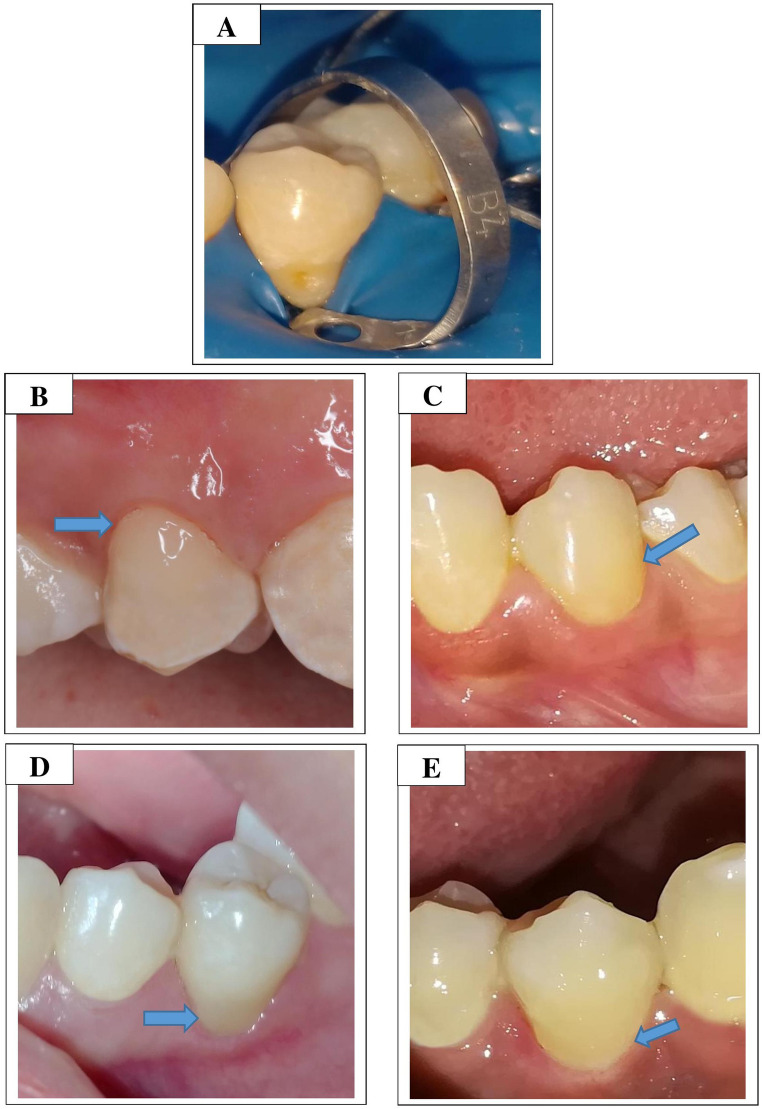
**A**: Representative image of the prepared cavity under rubber dam isolation. **B**: Final restoration after 2 years of follow-up for the bulk-fill composite bonded with a non–fluoride-releasing universal adhesive. **C**: Final restoration after 2 years of follow-up for the bulk-fill composite bonded with a fluoride-releasing universal adhesive. **D**: Final restoration after 2 years of follow-up for the ion-releasing composite bonded with a non–fluoride-releasing universal adhesive. **E**: Final restoration after 2 years of follow-up for the ion-releasing composite bonded with a fluoride-releasing universal adhesive

No statistically significant differences were observed in the ratings of all primary outcome FDI criteria across different assessment periods within the same restorative system group or among different restorative system groups within the same evaluation timeframe (*p* > 0.05) (Table 6; Fig. 2). The majority of scores for all criteria were rated as score 1. Postoperative sensitivity was the only criterion in which initial scores were worse than those recorded at subsequent evaluations; however, this difference was not statistically significant.

**Table 6:** Summary of FDI clinical criteria findings of different restorative systems’ groups over two years’ follow-up period

Restorative system		BF/F−				BF/F+				IR/F−				IR/F+			
Criteria	Score	0	6M	1Y	2Y	0	6M	1Y	2Y	0	6M	1Y	2Y	0	6M	1Y	2Y
Fractures and retention	**1**	28	28	28	28	30	30	30	30	30	30	30	30	29	29	29	29
Marginal adaptation	**1**	28	27	27	27	30	30	30	30	30	29	29	29	29	29	29	29
	**2**	0	1	1	1	0	0	0	0	0	1	1	1	0	0	0	0
Form and contour	**1**	28	28	28	28	30	30	30	30	30	30	30	30	29	29	29	29
Caries at restoration margin	**1**	28	28	28	28	30	30	30	30	30	30	30	30	29	29	29	29
Tooth defects at restoration margin	**1**	28	28	28	28	30	30	30	30	30	30	30	30	29	29	29	29
Postoperative hyper-sensitivity	**1**	25	28	28	28	26	30	30	30	28	30	30	30	26	29	29	29
	**2**	3	0	0	0	4	0	0	0	2	0	0	0	3	0	0	0
Marginal staining	**1**	28	28	28	28	30	30	30	30	30	30	29	29	29	29	29	29
	**2**	0	0	0	0	0	0	0	0	0	0	1	1	0	0	0	0
Surface luster and texture	**1**	28	28	28	28	30	30	30	30	30	30	29	29	29	27	27	27
	**2**	0	0	0	0	0	0	0	0	0	0	1	1	0	2	2	2
Patient’s view	**1**	26	28	28	28	28	30	30	30	27	30	30	30	28	29	29	29
	**2**	2	0	0	0	2	0	0	0	3	0	0	0	1	0	0	0

*BF* bulk-fill resin composite, *IR* ion-releasing composite, *F−* fluoride-free universal adhesive, *F+* fluoride-releasing universal adhesive

### Secondary outcomes findings

The outcomes for the secondary measures are presented in Table 6. For surface luster and texture as well as patient’s view, no statistically significant differences were identified between restorative systems within the same evaluation timeframe or among different follow-up assessments within the same restorative system (*p* > 0.05). Initial evaluations regarding patient’s view were worse than those observed at later follow-up periods; however, this difference did not reach statistical significance.

## Discussion

Despite the growing availability of ion-releasing and bioactive restorative materials, clinical evidence supporting their superiority over conventional resin composites—particularly when combined with fluoride-releasing adhesives—remains limited and inconsistent [16]. Most available data are derived from laboratory studies or clinical trials with heterogeneous cavity designs and operative protocols, limiting direct comparisons [27]. The present randomized clinical trial contributes standardized clinical evidence by directly comparing a bulk-fill resin composite and an ion-releasing composite in Class V carious cervical lesions, while simultaneously assessing the effect of fluoride-releasing versus fluoride-free universal adhesives over a two-year period using updated FDI criteria and periodontal parameters.

The two universal adhesives evaluated were one-step, mild formulations chosen to standardize the adhesive strategy across groups, with fluoride release as the primary variable [28]. Selective enamel etching followed contemporary recommendations for resin composites [29]. Although phosphoric acid etching can affect chemical interactions of functional monomers like 10-MDP with dentin, micromechanical interlocking and hybrid layer formation still occur, supporting stable adhesion [30]. With the standardized protocol, these effects were expected to be comparable across groups and unlikely to affect outcomes.

The ion-releasing composite was applied according to the manufacturer’s instructions. Although concerns have been raised regarding potential limitations in ion diffusion through adhesive layers, available evidence suggests that ion exchange may still occur depending on adhesive permeability [7, 31, 32]. As no clinical differences were observed in the present study, any such effects were unlikely to be clinically relevant within the two-year observation period.

Bulk-fill placement was intentionally selected for all groups to standardize the restorative technique. Class V cavities present unfavorable biomechanical conditions, including a high C-factor and cervical tooth flexure, which increase interfacial stress [33]. Both tested materials are designed for bulk placement and stress reduction, minimizing technique-related bias and allowing a more direct comparison of material performance [34].

The standardized cavity preparation protocol included selective beveling of the occlusal enamel margins only. Although enamel beveling has traditionally been advocated to enhance enamel bonding and marginal adaptation [35], contemporary biomimetic concepts emphasize that its clinical benefit is context-dependent rather than universally required [36]. Accordingly, beveling in the present study was limited to enamel-rich occlusal margins, while dentin or cementum margins were intentionally left unbeveled, in line with current evidence questioning the routine application of bevels in all restorative situations [36, 37].

The manufacturer of the ion-releasing composite allows either undercutting or beveling of enamel margins in Class V cavities [38]. Proximal margins were not beveled when the gingival margin extended into dentin or cementum to minimize the risk of adverse gingival healing associated with beveling in these situations [37].

All restorations were placed under rubber dam isolation to minimize contamination and procedural variability, which may contribute to improved restoration durability [29]. Both patients and examiners were blinded to the group assignments. While the restorative materials showed slight shade variations, these differences were minimal and did not allow examiners to determine the material used during follow-up assessments.

Based on the study results, there were no statistically significant differences in any of the primary or secondary outcomes among the different restorative systems during any of the evaluation periods, nor between the different evaluation periods within each restorative system. Therefore, both null hypotheses were accepted.

There were no statistically significant differences in periodontal parameters among the different restorative systems or across follow-up periods within the same system. Although specific cytotoxicity data are not available for the tested bulk-fill resin composite, the comparable periodontal outcomes observed between this material and the ion-releasing composite may be explained by several factors. Direct tissue contact was minimal, as subgingival margins were limited to 1 mm below the gingival margin, avoiding violation of the supracrestal tissue attachment and thereby preserving periodontal health without inducing pathological pocket depth or bone loss [39, 40]. In addition, careful finishing and polishing produced smooth restoration surfaces adjacent to the gingiva, which is critical for reducing bacterial adhesion and maintaining periodontal health [41]. The consistently good marginal sealing observed across all evaluation periods may have further contributed to the absence of intergroup differences. Moreover, the tested bulk-fill composite shares similar resin chemistry with other materials in the Tetric family, which have demonstrated biocompatibility comparable to ion-releasing composites in previous studies [42], supporting the safe subgingival placement of both materials from periodontal and clinical perspectives [43].

According to the findings of the present study, no statistically significant differences were observed either among the four restorative systems or within the same system at different follow-up intervals. Furthermore, none of the restorations exhibited failure or received scores of 4 or 5 in any of the assessed FDI criteria. This consistency in performance might be attributed to the relatively limited duration of clinical observation, which extended over only two years.

In this trial, no restorations fractured during the follow-up period. This outcome may be attributed to the favorable mechanical properties of the tested materials. The bulk-fill composite contains large pre-polymerized filler particles embedded within a polymerized organic matrix and exhibits a high filler loading, features associated with improved mechanical stability [44, 45]. Likewise, laboratory studies have shown that the tested ion-releasing composite demonstrates higher fracture toughness with minimal micro-cracking compared with conventional resin composites [46]. These properties likely contributed to the absence of restoration fractures, despite the challenging biomechanical environment of Class V cavities.

The non–fluoride-releasing universal adhesive contains the functional monomer 10-MDP, which chemically interacts with hydroxyapatite to form a stable nanolayered calcium–phosphate–MDP complex, enhancing interfacial integrity, long-term stability, and marginal adaptation [47]. The adhesive also includes Decandiol dimethacrylate (D3MA) and a methacrylated carboxylic acid polymer (MCAP), which improve compatibility with resin composites and promote chemical bonding to dental hard tissues through multiple carboxyl-mediated interactions with hydroxyapatite [45, 48]. Together, these compositional features contribute to a robust and durable adhesive interface.

The tested fluoride-releasing adhesive features an optimized formulation designed to enhance bonding stability, including reduced HEMA content, high-purity 10-MDP, and a hydrophilic acrylamide-based monomer that improves resin penetration while minimizing HEMA dependence [49]. The lower HEMA concentration, as reported by the manufacturer, is associated with reduced water absorption, improved polymerization efficiency, and enhanced long-term durability of the adhesive layer [49].

The ion-releasing composite exhibited favorable handling characteristics, as its low filler loading and high flow may enhance adaptation to gingival margins and irregular cavity walls [48]. Its low elastic modulus allows limited flexure with the tooth and absorption of polymerization and functional stresses, reducing marginal debonding and microleakage—particularly at cervical dentin margins subjected to high stress concentrations [50]. Similarly, the favorable performance of the bulk-fill resin composite may be attributed to its low polymerization stress, achieved through shrinkage-stress–relieving fillers and the presence of pre-polymerized filler particles, which contribute to a reduced elastic modulus [51].

The previously mentioned data regarding the adhesives and restorative materials used may explain the comparable marginal staining and marginal adaptation observed among the four restorative systems. Additionally, these factors may account for the absence of any detected recurrent or secondary caries in all cases at the end of the evaluation period. A previous systematic review also reported similar clinical outcomes when comparing the tested ion-releasing composite with traditional resin composites [52].

No differences in postoperative hypersensitivity or pulp vitality were observed among the restorations at baseline or during the follow-up periods. All cavities were of shallow to moderate depth, and no defects at the restoration margins were detected at any evaluation time point, likely reflecting the minimally invasive cavity preparations that preserved the remaining tooth structure. Likewise, no significant intergroup differences were found in surface texture or luster. The use of a multi-step finishing and polishing system may have contributed to these findings, as this approach has been shown to be the most effective for polishing bulk-fill resin composites in terms of surface roughness and microbial adhesion [53].

In the present trial, both an ion-releasing adhesive and an ion-releasing restorative material were used; however, no clinical advantage of ion release was detected. Schwendicke et al. [54] demonstrated that marginal leakage, rather than intrinsic material properties, is the primary driver of caries-like lesion development adjacent to restorations, indicating that effective marginal sealing can prevent lesion formation even in the absence of ion release. This finding helps explain the lack of measurable benefit from the fluoride-releasing components in the current study. Consistent with this, multiple systematic reviews and both in vitro and in vivo studies have identified marginal integrity as one of the strongest predictors of secondary caries, with well-adapted margins exhibiting significantly lower microleakage and caries incidence [55, 56].

Restorations with well-adapted, intact margins minimize bacterial ingress and demineralization at the restoration–tooth interface. Dye-penetration, micro-CT, and biofilm-challenge studies show that even small marginal gaps allow biofilm penetration and wall lesion formation [54, 57]. When margins are sealed, the cariostatic effect of fluoride is largely unnecessary, as the primary pathway for lesion initiation is blocked [16, 58]. Conversely, once marginal integrity is lost, fluoride and other ion release from modern adhesives and resin composites is limited, typically showing an initial burst followed by rapid decline. In vitro studies and clinical reviews indicate this brief ion release cannot counteract demineralization from persistent microleakage and biofilm activity [58–60].

Systematic reviews comparing ion-releasing materials—including bioactive composites and glass-ionomer restoratives—with conventional resin composites report mixed outcomes. Glass ionomers may provide superior preventive effects in high-caries-risk patients and primary dentition, but evidence for additional protection from contemporary resin-based ion-releasing or bioactive materials in well-sealed routine restorations is limited or inconsistent [16, 58, 61]. Overall, these findings highlight the paramount importance of a durable, well-adapted marginal seal. Proper adhesive application, sound cavity design, and careful operative technique remain the main determinants of long-term performance, with fluoride release offering secondary benefits in well-controlled clinical settings.

The findings of this study should be interpreted considering several limitations. The two-year follow-up may be insufficient to detect long-term adhesive degradation, sustained ion release, or later secondary caries. Use of rubber-dam isolation and a single experienced operator likely reduced technique-sensitive variability, potentially masking differences under routine clinical conditions. Additionally, participants were instructed to use fluoride-free toothpaste to standardize fluoride exposure and minimize potential confounding effects; however, this approach may have limited the cariostatic benefits for non-restored teeth. This approach was considered ethically acceptable due to the implementation of professional prophylaxis, reinforcement of oral hygiene measures, and close clinical monitoring; however, this aspect should be considered when extrapolating the findings to routine clinical practice. Future research should include extended follow-up periods to assess the long-term stability of these restorative systems and to determine whether late divergences in marginal integrity or caries prevention emerge over time.

## Conclusions

Within the limitations of this two-year randomized clinical trial, all four restorative systems showed comparable and favorable clinical performance in Class V carious lesions. Neither the use of a fluoride-releasing adhesive nor the incorporation of an ion-releasing restorative material resulted in additional clinical benefit over the evaluation period. The consistent outcomes across all groups suggest that, when appropriate adhesive protocols and careful restorative techniques are applied, both bulk-fill resin composites and ion-releasing composites can perform reliably in routine clinical practice.

## Supplementary Information

Below is the link to the electronic supplementary material.

Supplementary Material 1 (DOCX 13.8 KB)

Supplementary Material 2 (DOCX 15.1 KB)

## Data Availability

The datasets generated and/or analyzed during the current study are not publicly available as this part of doctoral dissertation but are available from the corresponding author upon reasonable request.
