# Supplementary material for: A two-year randomized clinical trial of bulk-fill and ion-releasing composites with universal adhesives in class V carious lesions
Source: Clin Oral Investig. 2026 Apr 11;30(5):172. doi: 10.1007/s00784-026-06852-5 (PMC13070072; doi:10.1007/s00784-026-06852-5)
Supplement: Supplementary file 1 — Supplementary Material 1 (DOCX 13.8 KB) [file 784_2026_6852_MOESM1_ESM.docx]

**Supplementary Table S1** Number of lesions according to sex and age of patients

| Characteristics of Patients | Number of Lesions |
| --- | --- |
| Sex |  |
| - Female | 81 |
| - Male | 59 |
| Age (years) |  |
| - 25-35 | 56 |
| - 36-45 | 84 |
