# Supplementary material for: A two-year randomized clinical trial of bulk-fill and ion-releasing composites with universal adhesives in class V carious lesions
Source: Clin Oral Investig. 2026 Apr 11;30(5):172. doi: 10.1007/s00784-026-06852-5 (PMC13070072; doi:10.1007/s00784-026-06852-5)
Supplement: Supplementary file 2 — Supplementary Material 2 (DOCX 15.1 KB) [file 784_2026_6852_MOESM2_ESM.docx]

| **Characteristics of restored tooth** | **BF / F−**  **(*n*=35)** | **BF / F+**  **(*n*=35)** | | **IR / F−**  **(*n*=35)** | **IR / F+**  **(*n*=35)** |
| --- | --- | --- | --- | --- | --- |
| **Number of restorations per patient** |  | | | | |
| One restoration | 21 | 17 | | 25 | 13 |
| Two restorations | 7 | 9 | | 5 | 11 |
|  | **Number of lesions** | | | | |
| **Teeth distribution** |  | | | | |
| - Premolars | 29 | 25 | | 31 | 27 |
| - Molars | 6 | 10 | | 4 | 8 |
| **Dental arch distribution** |  | | | | |
| - Upper | 13 | 8 | | 16 | 7 |
| - Lower | 22 | 27 | | 19 | 28 |
| **Type of tissue cervically** |  | | | | |
| - Enamel | 30 | 28 | | 31 | 27 |
| - Dentin | 5 | 7 | | 4 | 8 |
| **Approximation to the gingival margin (At the time of diagnosis)** |  | | | | |
| - Supragingival | 0 | 2 | 0 | | 3 |
| - Equigingival | 19 | 22 | 18 | | 16 |
| - Subgingival | 16 | 11 | 17 | | 16 |
| BF: bulk-fill resin composite; IR: ion-releasing composite; F−: fluoride-free universal adhesive; F+: fluoride-releasing universal adhesive | | | | | |

**Supplementary Table S2**. Characteristics of restored cavities
